# Multi-Drug/Gene NASH Therapy Delivery and Selective Hyperspectral NIR Imaging Using Chirality-Sorted Single-Walled Carbon Nanotubes

**DOI:** 10.3390/cancers11081175

**Published:** 2019-08-14

**Authors:** Md. Tanvir Hasan, Elizabeth Campbell, Olga Sizova, Veronica Lyle, Giridhar Akkaraju, D. Lynn Kirkpatrick, Anton V. Naumov

**Affiliations:** 1 Department of Physics and Astronomy, Texas Christian University, TCU Box 298840, Fort Worth, TX 76129, USA; 2 The University of Texas MD Anderson Cancer Center, 1515 Holcombe Blvd, Houston, TX 77030, USA; 3 Department of Biology, Texas Christian University, 2955 South University Drive, Fort Worth, TX 76129, USA; 4 Ensysce Biosciences, 3210 Merryfield Row, San Diego, CA 92121, USA

**Keywords:** single-walled carbon nanotubes, chirality separation, NASH, drug-gene delivery, near IR hyperspectral imaging

## Abstract

Single-walled carbon nanotubes (SWCNTs) can serve as drug delivery/biological imaging agents, as they exhibit intrinsic fluorescence in the near-infrared, allowing for deeper tissue imaging while providing therapeutic transport. In this work, CoMoCAT (Cobalt Molybdenum Catalyst) SWCNTs, chirality-sorted by aqueous two-phase extraction, are utilized for the first time to deliver a drug/gene combination therapy and image each therapeutic component separately via chirality-specific SWCNT fluorescence. Each of (7,5) and (7,6) sorted SWCNTs were non-covalently loaded with their specific payload: the PI3 kinase inhibitor targeting liver fibrosis or CCR5 siRNA targeting inflammatory pathways with the goal of addressing these processes in nonalcoholic steatohepatitis (NASH), ultimately to prevent its progression to hepatocellular carcinoma. PX-866-(7,5) SWCNTs and siRNA-(7,6) SWCNTs were each imaged via characteristic SWCNT emission at 1024/1120 nm in HepG2 and HeLa cells by hyperspectral fluorescence microscopy. Wavelength-resolved imaging verified the intracellular transport of each SWCNT chirality and drug release. The therapeutic efficacy of each formulation was further demonstrated by the dose-dependent cytotoxicity of SWCNT-bound PX-866 and >90% knockdown of CCR5 expression with SWCNT/siRNA transfection. This study verifies the feasibility of utilizing chirality-sorted SWCNTs for the delivery and component-specific imaging of combination therapies, also suggesting a novel nanotherapeutic approach for addressing the progressions of NASH to hepatocellular carcinoma.

## 1. Introduction

The use of nanomaterials as gene/drug delivery agents has increased significantly over the past few years, owing to their capability of delivering either water-insoluble or unstable drugs or degradable gene therapeutics. Several categories of nanocarriers have been developed/utilized thus far, including quantum dots [1,2,3,4], PLGA-PEG (polylactic acid-co-glycolic acid-polyethylene glycol) nanoparticles [5], liposomes [6], self-emulsifying drug delivery systems (SEDDSs) [7], cyclodextrins [8], gold nanoparticles [9], and carbon nanotubes [10,11] as delivery vehicles or diagnostic tools. Among those, single-walled carbon nanotubes (SWCNTs) showed highly promising results for gene/drug delivery coupled with in vitro as well as in vivo imaging [12]. Their quasi-one-dimensional hydrophobic platform aids cellular internalization and the non-covalent or covalent attachment of active agents and targeting moieties [13]. At the same time, the intrinsic photostable fluorescence emission of SWCNTs in NIR (near-infrared) I and II regions with reduced scattering and autofluorescence backgrounds allows imaging through the layers of biological tissue [14,15]. As molecular transporters, SWCNTs can shuttle payloads, including drug molecules [16], proteins [17], DNA [18], and RNA [19] into biological cells and tissues. Functionalized with drugs and targeting agents covalently [20] or non-covalently by π-π stacking [13,21], SWCNTs can provide reduced toxicity [22], greater biological activity [16], accumulation in the liver when formulated [10], and controlled drug release [23]. Due to characteristic NIR SWCNT fluorescent emission, their location can be imaged to confirm the payload delivery. As near-infrared exhibits significantly enhanced tissue penetration and lower scattering, it provides unique promise in the in vivo imaging of shallow targets. Although it has been shown that SWCNTs can be used for the delivery of drugs [11,24], cancer therapeutic siRNA oligos [10,11], and imaging agents [25,26], their capability for multidrug therapy and imaging has been underexplored to date, hampering their advancement to the successful treatment of complex conditions. Additionally, the ability to follow chirally separated SWCNTs by imaging allows one to confirm that each component in multidrug therapies reaches the desired tissue of interest.

SWCNT formulation is known to accumulate in the liver [10], which offers the potential for the treatment of liver diseases including nonalcoholic steatohepatitis (NASH) and its progression into hepatocellular carcinoma (HCC). Thus, in this work, NASH was chosen as a feasible treatment target to demonstrate the imaging/drug delivery capabilities of SWCNTs. NASH is a non-curable condition present in 6–8% of adults in the US. It accounts for a large number of cases of cirrhosis and can progress to HCC, leading to 75% of all liver cancer—the third leading cause of cancer-related deaths. The transformation of NASH into HCC is known to be mediated by fibrosis [27] progressing in over 30% of NASH patients, and inflammatory response with the involvement of a variety of cytokines [28]. Due to the complexity of this condition involving both inflammation and fibrosis, multifactor treatment strategies are required.

Current molecular therapy approaches are often restricted by drug resistance and the inability to target multiple factors [29]. These challenges can be addressed by combination treatments involving multidrug approaches to surmount drug resistance, decrease treatment doses, and affect multiple therapeutic targets. Although effective in treatment [30,31,32], combination therapies suffer from non-specific toxicity [33,34], difficulties in assessing the adverse effects of each drug separately, and the lack of image-guided capabilities [35]. On the other hand, combination gene [36,37,38] or drug/gene [39] therapies can circumvent the issue of non-specific toxicity due to the target-specific effects of gene therapeutics while providing effective routes to treatment. However, due to the short lifetime of DNA/RNA oligonucleotides in the body, additional delivery mechanisms are required [40]. 

Here we explore a therapeutic platform with the capacity to address both inflammation and fibrosis pathways of NASH via combination drug/gene therapy. The therapeutic approach used an siRNA target that has been shown to reduce the inflammatory cytokines that lead to liver fibrosis [41], and a small-molecule PI3 kinase inhibitor, PX-866 [28], that has been shown to reduce tissue fibrosis in vivo. Fibrosis is known to increase the risk of HCC by 25 times [42], as it leads initially to liver cirrhosis and subsequently develops into HCC. In this progression, activated hepatic stellate cells (aHSCs) responsible for fibrosis development are often described as pericytes for angiogenesis and vascular remodeling in the liver [43]. Fibrosis mediated by HSCs is associated with the effects of inflammation, as multiple inflammatory cytokines are known to elicit further activation of HSCs [44]. Although the entire process is not fully understood, fibrotic cytokine release (i.e., TGF-β, sonic hedgehog, and TNF-α) in the course of NASH is believed to contribute to the progression of the latter through the fibrotic stage and cirrhosis to HCC [28]. Therefore, developing delivery and tracking through imaging modules for therapeutic entities that address both fibrosis and inflammation in NASH could be an important step toward mitigating the transformation of NASH into HCC. 

Hepatic inflammation can be suppressed by gene therapies interfering with cytokine activation, including several siRNA sequences against the protein. CCR5 (aka RANTES) siRNA is well-known for its anti-inflammatory effects [45,46,47], and could be a potential key to the reduction of inflammation in the liver. Among effective fibrotic drugs, a PI3 kinase inhibitor, PX-866, was chosen for two reasons: (1) PX-866 was previously shown to reduce fibrosis in the lungs [48]; (2) PX-866 has been evaluated in clinical trials, its safety profile is understood, and it had shown some clinical benefit against solid-tumor cancers [49]. To reduce off-target effects of PX-866 and protect siRNA from enzymatic degradation in the blood, these therapies could benefit from a delivery vehicle that will direct their transport to the liver. Additionally, to allow confirmation of the delivery of each therapeutic entity by the SWCNTs, the capability of image-tracking chiral SWCNTs is advantageous.

In the present work, chiral SWCNTs perform the critical function of delivery/imaging agents capable of protecting siRNA from enzymatic degradation in circulation, and protecting healthy tissue from the off-target effects of PX-866 by focusing this delivery to the liver tissue. While using SWCNTs of one select chirality for the delivery of either siRNA sequence or a drug payload, chirality-specific SWCNT fluorescence [50] in the NIR can be utilized to image the location and delivery pathways of the drug or gene separately. 

The goal of this work was to assess the capabilities of SWCNTs for the non-covalent delivery and imaging of combination therapeutics such as PX-866 and siCRR5, each attached to SWCNTs of a particular chirality. The efficacy of each payload was evaluated using hepatocellular carcinoma cells (HepG2) while NIR hyperspectral imaging was used to confirm the location and SWCNT-mediated delivery of each therapeutic separately. Since SWCNTs are produced as a mixture of chiralities, several separation strategies have been developed to isolate single chirality fractions, including gel chromatography [51], density-gradient ultracentrifugation [52], free-solution electrophoresis [53], aqueous two-phase extraction (ATPE) method [54], etc. ATPE is well-known and widely used for its high yield with maximum purity, low-cost surfactants, high production scalability, and availability of instrumentation required for separation in every laboratory. Therefore, in order to develop scalable and affordable combination therapy platforms, in this study single-chirality SWCNTs were separated by a modified ATPE method [54]. We isolated (7,5) and (7,6) chiral SWCNTs from raw CoMoCAT (Cobalt Molybdenum Catalyst) SWCNT samples, as those chiralities exhibit spectrally well-separated emission at 1035 nm and 1130 nm, respectively. NIR hyperspectral imaging was used to separately confirm the internalization of (7,5) and (7,6) chiral nanotubes, ensuring the delivery of drug and gene inside the HepG2 cells. Overall, this work explores the feasibility of using single-chirality SWCNTs as efficient imaging and delivery vehicles. Eventually, the utilization of such chiral SWCNTs may lead to the development of a unique image-guided multimodal therapy addressing several therapeutic targets. Here we particularly explore the possibility of targeting both inflammation and fibrosis, which facilitate the progression of NASH to HCC.

## 2. Results and Discussion

Since raw SWCNT samples contain nanotubes of different chiralities as well as SWCNT aggregates that are non-emissive and unsuitable for drug delivery, prior to sorting (7,5) and (7,6) chiral nanotubes, raw CoMoCAT SWCNT samples initially underwent aggregate depletion via 180 min centrifugation of sodium deoxycholate (DOC)-dispersed SWCNTs at 21,380× *g*. This rigorous procedure resulted in the sedimentation of SWCNT aggregates with higher specific gravity than the individually wrapped SWCNTs. In the aqueous two-phase extraction (ATPE) method, centrifuged SWCNTs with constant DOC concentration were combined in a PEG–dextran two-phase system with a variety of sodium dodecyl sulfate (SDS) concentrations, yielding the separation of chiralities from the dextran-enriched bottom to the PEG-enriched top phase. The (7,5) and (7,6) chiral nanotubes were separated at 3 and 4 mg/mL of SDS, respectively allowing a substantial degree of control over top/bottom phase chirality composition, evident from their respective fluorescence and absorbance spectra (Supporting Information Figures S1 and S2) that were pronouncedly different from each other and from the spectra of the parent samples.

**Figure 1:**
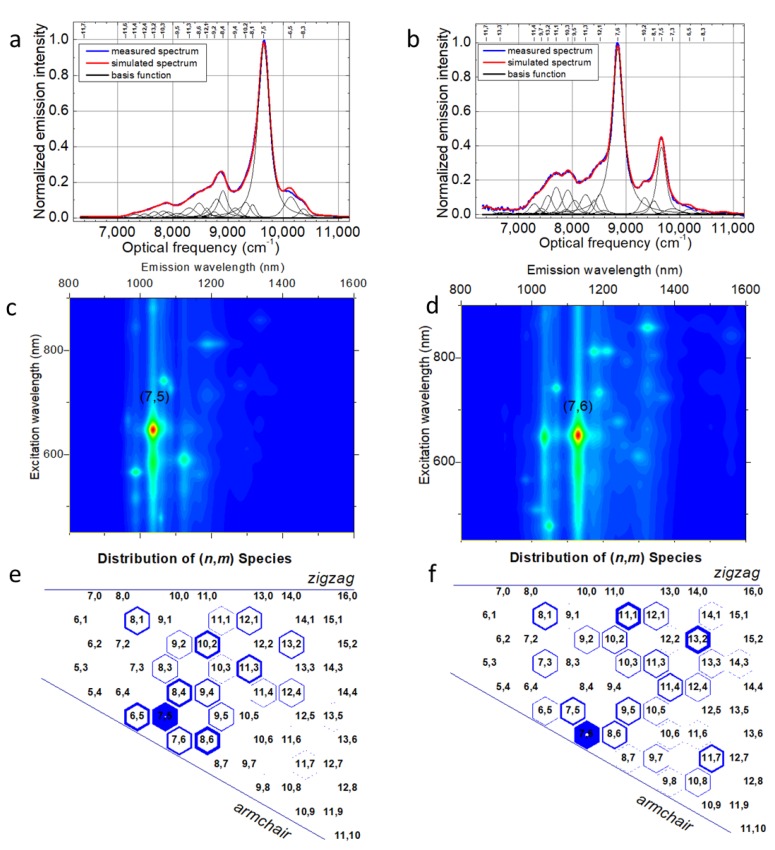
Measured and simulated fluorescence spectra of separated (**a**) (7,5), (**b**) (7,6) chiral single-walled carbon nanotubes (SWCNTs). The corresponding generated photoluminescence–excitation contour plot of (**c**) (7,5), (**d**) (7,6) sorted fractions. Graphene sheet representing the distribution of the emissive species in the respective (**e**) (7,5) and (**f**) (7,6) enriched sorted SWCNT samples. The blue-filled portion of hexagons represent the relative abundance of the species.

Simulation of fluorescence spectra of (7,5) and (7,6) sorted fractions with single SWCNT chirality Lorentzian emission profiles (Figure 1a,b) using the Applied Nanofluorescence Nanospectralyzer fitting routine allows for quantitative assessment of the sample optical properties and composition, yielding calculated excitation–emission maps (Figure 1c,d) and relative abundances (Figure 1e,f). The spectral fitting process used in this work was based mainly on adjusting the expected widths and positions of theoretical fluorescence peaks from a variety of semiconducting SWCNT chiralities to simulate experimental emission spectra collected with four excitation wavelengths (532, 637, 671, and 782 nm), aiming for a perfect match between the simulated and measured spectra (Figure 1a,b).

**Figure 2:**
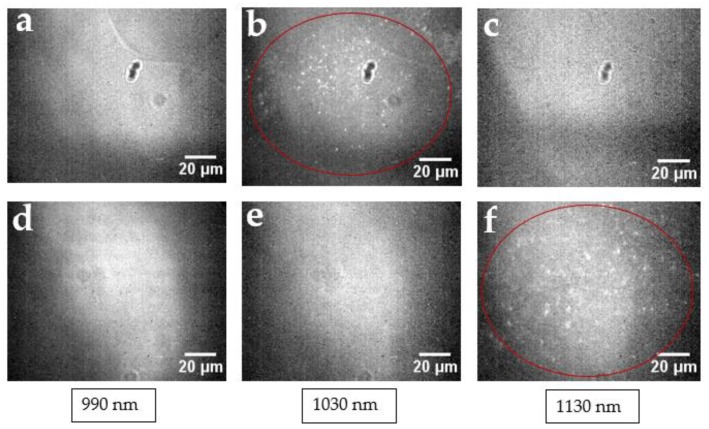
Near-infrared (NIR) hyperspectral images of (7,5) sorted SWCNTs at (**a**) 990, (**b**) 1030, and (**c**) 1130 nm; and (7,6) sorted SWCNTs at (**d**) 990, (**e**) 1030, and (**f**) 1130 nm. SWCNT fluorescence is only observed at the emission wavelengths of the corresponding sorted SWCNTs.

Based on the chirality abundances, calculated from the weight of individual chirality contributions to the experimental spectra and reflected in the distribution of (n,m) species (Figure 1e,f), substantial chirality enrichment was achieved in both sorted fractions (either (7,5) or (7,6)), with yields up to 40%. Although not overwhelming, this enrichment left other chiralities with only 1–5% contribution. In order to verify that this degree of separation is sufficient to monitor mainly SWCNTs of a single chirality microscopically, we examined the emission from (7,5) and (7,6) chirally sorted SWCNTs via NIR hyperspectral imaging at particular wavelengths corresponding to (7,3) (990 nm), (7,5) (1024 nm), and (7,6) (1120 nm) SWCNT chirality emission [55]. (7,3) SWCNT emission was dominant in the unsorted sample (Supporting Information Figures S1 and S2) and thus could be a significant contaminant in both fractions. However wavelength-resolved microscopy images show that the (7,5)-sorted fraction exhibited emission only at 1030 nm (Figure 2a–c), corresponding only to (7,5) SWCNTs, whereas the (7,6) fraction only showed observable SWCNT emission at 1130 nm (Figure 2d–f), corresponding to (7,6) SWCNTs. No substantial cross-contamination or contamination from (7,3) SWCNTs was observed. Furthermore, no observable contamination of the sorted sample by other SWCNT chiralities was detected by hyperspectral microscopy as we scanned the emission from 950 to 1350 nm with the step of 10 nm. This indicates that the achieved degree of separation with minor percentages of contaminants of each chirality was sufficient for hyperspectral microscopy imaging.

**Figure 3:**
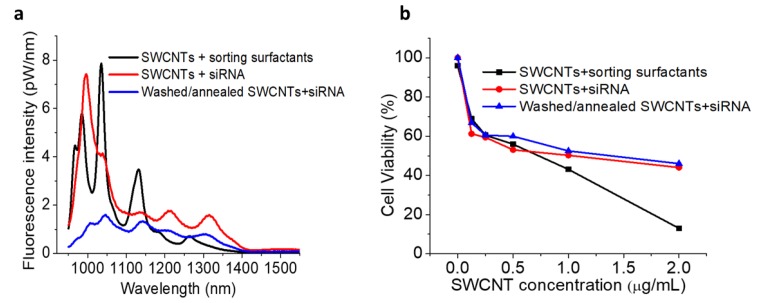
(**a**) Fluorescence spectra of raw SWCNTs dispersed with sorting surfactants, raw SWCNTs dispersed with siRNA, and SWCNTs washed/annealed for surfactant removal and re-dispersed with siRNA showing similar peak positions for raw SWCNTs dispersed with siRNA and washed/annealed SWCNTs dispersed with siRNA. (**b**) Cell viability of HepG2 cells subject to SWCNTs dispersed with sorting surfactants, raw SWCNTs dispersed with siRNA, and SWCNTs washed/annealed for surfactant removal and re-dispersed with siRNA.

One of the main drawbacks of utilizing chirality-sorted SWCNTs has always been the high toxicity of sorting surfactants. In order to overcome this issue, we performed repeated multi-step centrifugal filtration (washing) with methanol/ethanol/DI water followed by the annealing of sorted SWCNTs at 200 °C for 1 h to remove the additional surfactants from the sorted SWCNTs. The annealed SWCNT samples were cooled down to room temperature before any further processing. The degree of surfactant removal was first verified spectroscopically by comparing the fluorescence spectral features of processed surfactant-purified samples: SWCNTs washed/annealed re-dispersed with EGFR siRNA and the spectra of raw SWCNTs dispersed directly with the same EGFR siRNA (Figure 3a). siRNA was chosen as it complexes non-covalently with SWCNTs and is known to form stable dispersions. Since surfactant wrapping induces observable fluorescence shifts specific to each surfactant [56,57] that are substantially different for bile salts and nucleic acids [58], assessing shifts in the positions of major peaks allows the qualitative removal of surfactant. Here, for the convenience of comparing multiple chirality peaks, we washed/annealed an unsorted SWCNT fraction, but with all the separation surfactants that are regularly present in the sorted samples. Although a starting SWCNT sample which included sorting surfactants (PEG/dextran/SDS) exhibited major emission peaks at ca. 966, 986, 1035, 1130, 1185, and 1265 nm (Figure 3a—black line), upon surfactant removal processing and redispersion with siRNA, major emissive features were observed at 1004, 1046, 1141, 1212, and 1308 nm (Figure 3a—blue line). Substantial shifts in the spectral positions (i.e., 11–18 nm shifts for 986, 1035, and 1130 nm peaks, a 27 nm shift for the 1185 nm peak, and a 43 nm shift for the 1265 nm peak), along with suppression of the 966 feature, indicate a significant change in the dielectric environment of SWCNTs that may have occurred due to the surfactant removal and replacement with siRNA. Furthermore, new peak positions appeared to be close to those of raw SWCNTs dispersed with siRNA (Figure 3a—red curve) that displayed a weak shoulder at 974 nm along with the most prominent emission features at ca. 996, 1041, 1141, 1213, and 1315 nm (comparison of peak positions listed in Supporting Information Figure S3). The processed SWCNTs were re-dispersed, and yielded substantial fluorescence emission with characteristic spectra indicating that the SWCNTs were individualized, while the broadening could arise from only a loose aggregation. Despite the difference in relative peak intensities affected by some aggregation accompanying the process of surfactant washing, substantial surfactant-removal-facilitated shifts of emission peaks toward those of siRNA-dispersed raw SWCNTs suggest a significant degree of surfactant depletion. This may reduce the cytotoxicity of sorted SWCNTs, making them as suitable for biological applications as raw SWCNTs dispersed by siRNA. It was also observed that SWCNTs’ characteristic emissions were not affected by thermal annealing, suggesting the preservation of SWCNTs’ fluorescence properties due to the thermal annealing at 200 °C for 1 h (Supporting Information Figure S4). Additionally, a comparative fluorescence study was performed between before and after centrifuged washed/annealed SWCNTs + siRNA sample, exhibiting a slight insignificant change of photoluminescence intensity (Supporting Information Figure S5). 

A MTT cytotoxicity assay of centrifugally filtrated and thermally annealed SWCNTs further helped to assess if any residual surfactants could add to the toxicity profile of the formulation. Confirming the findings derived from spectral position matching, MTT assays showed that washed/annealed SWCNTs had the same or lower cytotoxicity than the raw SWCNTs dispersed in siRNA (Figure 3b). The significantly lowered cell viability found in the assessment of the toxic profile of the parent unsorted SWCNT sample with all separation surfactants presents the benefit of surfactant removal. Although this does not verify complete removal of surfactants, it indicates that washed/annealed SWCNTs are not more toxic than the raw SWCNTs, minimizing the toxicity contribution of sorting surfactants. Further decrease in toxicity of SWCNT/siRNA formulation could be achieved by masking it with DSPE-PEG 5000 [10], which was used for all in vitro studies in this work. 

**Figure 4:**
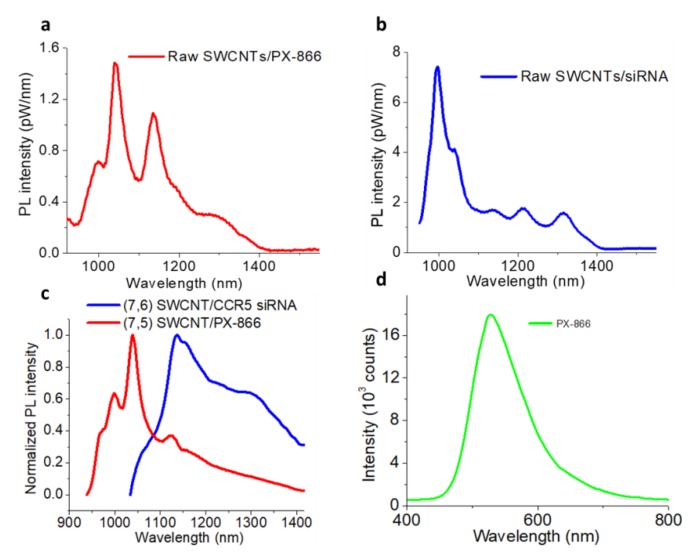
Emission spectra of raw CoMoCAT SWCNTs dispersed with (**a**) PX-866, (**b**) siRNA. Emission spectra of (**c**) (7,5) and (7,6) sorted washed/annealed SWCNTs dispersed with PX-866 and CCR5 siRNA, respectively. (**d**) Visible emission spectrum of PX-866 with 400 nm excitation.

Similar to siRNA, SWCNTs can non-covalently complex with another therapeutic (i.e., PX-866) that was used in this work. Upon ultrasonic processing, raw/unsorted SWCNTs showed a stable dispersion in an aqueous solution of PX-866, as well as distinct fluorescence emission (Figure 4a). Sorted, washed, and annealed SWCNT samples of (7,5) chirality could also be dispersed with PX-866 alone, showing distinct emission features corresponding to (7,5) SWCNTs (Figure 4c). Similarly, with only siRNA dispersion, raw SWCNTs (Figure 4b) and sorted washed/burned SWCNTs ((7,6) chirality) (Figure 4c) also formed stable emissive dispersions. In fact, corresponding fluorescence (Figure 4c) and absorbance (Supporting Information Figure S6a,b) spectra of (7,5) SWCNT/PX-866 and (7,6) SWCNT/siCCR5 fractions still showed the major features of the sorted SWCNT chiralities. Interestingly, PX-866 on its own showed emission in green with 400 nm excitation (Figure 4d) that was quenched when the drug was loaded on the SWCNTs (Supporting Information Figure S7). This feature was used to locate/ensure the delivery of the drug in HepG2 cells as it was released from SWCNTs and the emission was restored.

In order to ensure the lower cytotoxicity, improved stability, and in vivo compatibility of the formulations for future studies, both SWCNTs dispersed with PX866 and siCCR5 were additionally coated with DSPE-PEG-5000 (at 1600 µM) via ultrasonic processing. Aggregates that were not fully dispersed were further removed by centrifugal processing at 16,000× *g* for 5 min, while the excess of siRNA or PX-866, as well as DSPE-PEG-5000, was centrifugally filtered with 100-kDa molecular cutoff filters, leaving only drug or gene/SWCNT complexes in the solution. TEM images of the final samples verify substantial SWCNT coating (Supporting Information Figure S8).

Following successful separation, removal of the sorting surfactants and non-covalent attachment of the drugs and DSPE-PEG-5000, we further assessed the capability of (7,5) and (7,6) SWCNTs to trace the delivery of the drug and gene intracellularly. Since combination therapy is envisioned for NASH, sorted (7,5) and (7,6) SWCNTs complexed with PX-866 and CCR5 siRNA were respectively combined in one suspension in equal proportions based on SWCNT concentrations and introduced to HepG2 cells. After up to 3 h incubation, cells were washed twice with PBS (phosphate-buffered saline) solution to remove any extracellular SWCNTs, and only those that were internalized were imaged. Among 0.5, 1, and 3 h incubation times tested, the highest intracellular emission was assessed at 3 h post transfection which was also found to be one of the optimal time points in the previous works [10,59,60]. 

However, the efficiency/maximum intracellular emission and cellular uptake may vary with cell types, SWCNT length, and SWCNT functionalization. For example, Sekiyama et al. [61] used oxygen-doped SWCNTs/PEG to perform the intracellular imaging in cultured murine cancer cells (Colon-26) showing no emission/uptake up to 1 day, but started showing/increasing internalization from day 3 to day 7. Additionally, Mao et al. [62] studied the cellular uptake and distribution of collagen-functionalized SWCNTs in bovine articular chondrocytes (BACs), showing longer retention in cells for more than one week. The cellular uptake of SWCNTs was previously hypothesized to occur via nano-spearing of the cell membrane [63,64], explained by needle-like hydrophobic SWCNT structure, while endocytosis [65,66] is currently deemed as a more plausible SWCNT entry pathway and is considered as a major internalization mechanism in the current work.

**Figure 5:**
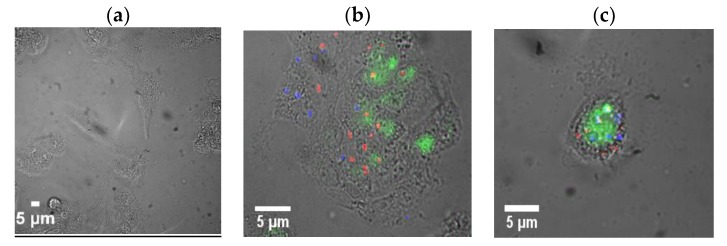
Brightfield/fluorescence overlay images of (**a**) untreated control HepG2 cells and (**b**,**c**) cellular uptake of (7,5) sorted SWCNTs imaged at 1030 nm (red), (7,6) sorted SWCNTs imaged at 1130 nm (blue), and PX-866 imaged at 535 nm (green) after the intracellular release.

Herein, in vitro fluorescence imaging was accomplished with custom microscopy setup involving an inverted microscope coupled to two visible (Hamamatsu Image EMCCD) and near-infrared (InGaAs Xenics Xeva) cameras, allowing for filtered emission detection in the visible and spectrally resolved imaging in the near-infrared enabled by a Photon etc. NIR hyperspectral imager. In that configuration, wavelength-resolved images were recorded in the visible range with a lamp and in the NIR with 637 nm laser excitation. This allowed the imaging of SWCNTs specifically at 1030 (Supporting Information Figure S9b) and 1130 nm (Supporting Information Figure S9c), corresponding to the emission wavelengths of sorted chiralities, while PX-866 was imaged in the visible with a 532 nm emission filter. Confirming the sufficient degree of chirality separation, no emission in the NIR was observed outside the aforementioned spectral regions. However, at 1030 and 1130 nm, substantial SWCNT fluorescence was observed within the cells (Supporting Information Figure S10), indicating the successful internalization of both formulations with no emission detected outside the cells. Due to spectrally resolved imaging, no autofluorescence was detected in the non-treatment control. The overlays of the fluorescence images of two chiral SWCNT fractions false-colored in red (for (7,5) SWCNT emission) and blue (for (7,6) SWCNT emission) (Figure 5) verify the capability of tracking each therapeutic separately, as one could pinpoint the internalized SWCNTs or their clusters within HepG2 cells. Additionally, the release of PX-866 could be assessed, as the released drug was no longer quenched by complexation to the SWCNTs: its fluorescence was also observed in the cells and was delocalized from its delivery vehicles while the extracellular PX-866 was removed by washing. PX-866 imaging settings including integration time and excitation lamp intensity were chosen such that they yielded no autofluorescence from non-treatment control cells (Figure 5a). Additionally, the intracellular release of PX-866 was tracked qualitatively with different incubation times (0, 1, 4.5, 12 h) (Supporting Information Figure S11), yielding maximum release at the 3 h time point, also suggesting that 3 h is the best possible incubation time for the intracellular imaging. A separate cellular internalization study was performed in HeLa (cervical cancer) cells, showing a brighter intracellular SWCNT emission (Supporting Information Figure S12) and indicating a substantial uptake capability of SWCNT/siRNA hybrids by several cancer cell lines.

**Figure 6:**
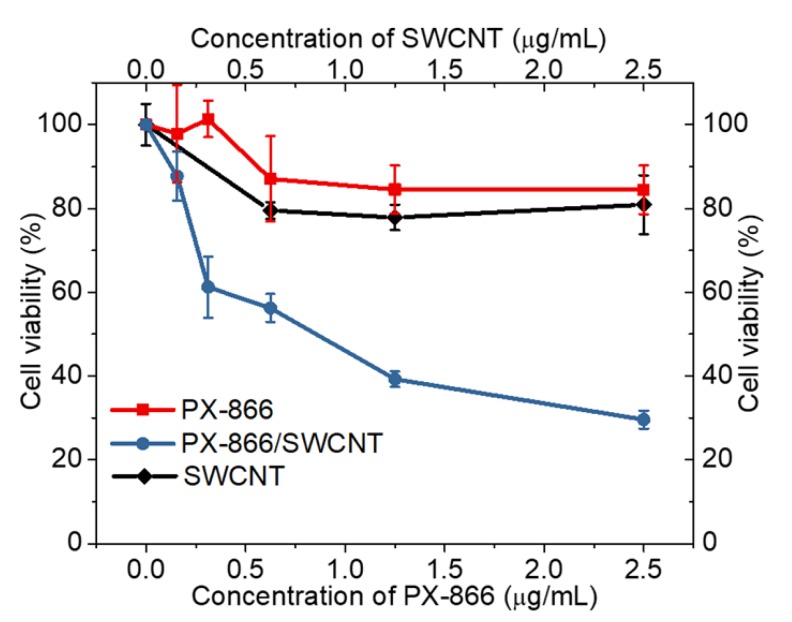
MTT assay cell viability of HepG2 cells treated with either SWCNTs, PX-866, or SWCNT/PX866 conjugates.

The efficacy of the delivered and imaged therapeutics was assessed separately for each drug and gene. The biocompatibility of SWCNTs in DSPE-PEG5000 coating used in these efficacy studies was verified both in HepG2 (Figure 6) and HeLa (Supporting Information Figure S13) cells, indicating that at imaging and treatment concentrations SWCNTs do not exhibit substantial toxicity to several cell lines. Due to the complexity of testing antifibrotic properties of PX-866 in vitro, its efficacy was assessed through the toxic response to HepG2 cancer cells. A comparative MTT cytotoxicity assay was used to assess the toxic effect of PX-866 alone or delivered by the SWCNTs (Figure 6). When formulated with SWCNTs, PX-866 showed more significant cytotoxic (Figure 6) effect, increased by the factor of ~2.8 at 2.5 µg/mL likely due to improved transport with the nanomaterial delivery vehicle, generally known to enhance the efficacy of delivered therapeutics [67,68,69]. The antifibrotic effect of the drug leads to apparent toxicity that is best analyzed in the cancer cells. The toxicity added by SWCNTs alone cannot be responsible for that increase, as those at 2.5 µg/mL equivalent to 2.5 µg/mL PX-866 concentrations showed only a small toxic response, with cell viability above 80%. For the cytotoxicity testing, the SWCNT/PX-866 conjugation was accomplished using the concentration ratio of 1:1 for SWCNT and PX-866. This yielded a stable SWCNT dispersion via non-covalent complexation with the drug.

**Figure 7:**
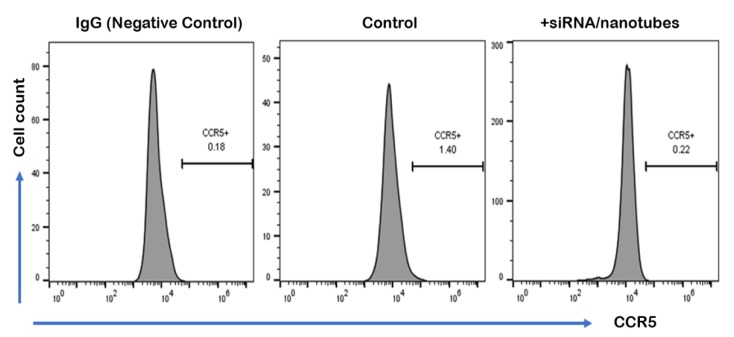
Downregulation of CCR5 in HepG2 cells by siRNA/nanotubes complex. CCR5 expression was detected by flow cytometry after nanotubes-delivered siRNA-mediated knockdown for 48 h. IgG staining was used for isotype control (**left**). Control indicated natural expression of CCR5 in HepG2 cells without any treatment (**middle**) compared to CCR5-targeting siRNA/nanotubes treatment (**right**).

The efficacy of SWCNT/siCCR5 formulation was evaluated by assessing the CCR5 siRNA-mediated knockdown in HepG2 cells after 48 h transfection via flow cytometry, as CCR5 is known to be expressed in HepG2 cells [70]. SWCNTs-formulated siRNA transfection showed much lower expression of chemokine receptor type 5 (CCR5) as compared to the natural expression exhibited in the control sample (Figure 7), indicating substantial (over 90%) knockdown above or comparable to that regularly achieved by lyposomally delivered siRNA [18,71,72]. 

It is evident that SWCNTs also facilitated siRNA transfection, as it does not transfect mammalian cells on its own [73,74,75]. IgG (immunoglobulin G) antibody was used as isotype control, to help differentiate the non-specific background signal from specific antibody signal. Overall, this study verifies the efficacy of SWCNT/siCCR5 formulation and suggests SWCNTs as a promising gene-silencing carrier for NASH therapeutics.

It is also noteworthy that SWCNTs have the ability to protect the probe (siRNA) from degradation in blood circulation, because: (1) they offer only a small window for nucleases/proteins to degrade siRNA bound to the SWCNT surface; and (2) conjugation of SWCNT/siRNA may form an unusual RNA structure which helps to disguise the siRNA from enzyme binding sites [76]. Additionally, siRNA coating can prevent blood proteins from binding to the SWCNT surface [10], reducing the protein corona formation. The delivered therapeutics were analyzed separately in vitro as the effects of PX-866 would interfere with the determination of protein knockdown. However, in vivo they are expected to perform synergistically against NASH-induced inflammation and fibrosis. The present work shows the potential of chirality-sorted SWCNTs for delivery, separate NIR fluorescence imaging, and increase in the efficacy of combination drug/gene therapy aimed to address inflammation and fibrosis in NASH, which is necessary for the further application of SWCNT-mediated combination therapy for NASH in vivo. Further NASH animal model studies will lead to the direct assessment of the antifibrotic potential of the SWCNT-delivered PX-866 via PCR analysis of TGF-B1, B2 expression, together with the assessment of the synergistic effects of the nanotherapeutics developed here.

## 3. Experimental Methods and Procedures

### 3.1. Sample Preparation

The suspensions of SWCNTs (CoMoCAT, (7,6) chirality, ≥77% carbon as SWCNT) were prepared by dispersing 1.5 mg of SWCNTs in 1 mg/mL sodium deoxycholate (DOC) aqueous solution. The samples were further processed via direct probe ultrasonic treatment (QSonica, Q55) for 60 min at 33 W in an ice bath. The suspension was further centrifuged (Southwest Science, D3024) for 180 min at 21,380× *g* followed by the removal of SWCNT aggregates into the decant and collecting the supernatant only. The collected SWCNTs were used for the chirality sorting. In order to create two phases, 0.25 g/mL aqueous solution of PEG (MW—6 kDa) and 0.25 g/mL aqueous solution of dextran (MW—75 kDa) were used to produce the stock solution. After that, a ratio of 0.4:0.28:0.28:0.04 (SWCNTs:PEG:dextran:water) was maintained to prepare the final sample for the separation. Sodium dodecyl sulfate (SDS) was added to this final suspension with a variation in concentration from 1 to 7 mg/mL. The targeted (7,5) and (7,6) chiral tubes were separated in the top phase at 3 and 4 mg/mL SDS concentration. 

In order to remove sorting surfactants, we performed repeated centrifugal filtration (Amicon Ultra 0.5 mL; 100,000 MWCO filter) using methanol (to condense the samples first) for five times followed by 15 times filtration with ethanol to wash the remainders of the surfactants from the separated SWCNTs. As surfactants were washed through the filter pores, SWCNTs remained on the filter. Washed samples were collected and further processed for thermal annealing at 200 °C for one hour in the mechanical convection oven (precision-18EM laboratory oven). Processed (7,5), (7,6) chiral SWCNTs were then collected and dispersed with PX-866 and CCR5 siRNA (Biolegend, San Diego, CA, USA, Cat#359105) in aqueous suspension, respectively. To assure non-covalent complexation, 0.3 mg/mL of SWCNTs and 0.5 µg/mL of siRNA in nuclease-free water or 0.3 mg/mL of PX-866 in DI water were mixed and subjected to ultrasonic treatment using a Covaris S2 (SN001263) ultrasonic disperser at 70 W for 2 min, which allowed avoidance of contact with the non-sterile probe. Both siRNA and PX-866 were used at concentrations substantially below saturation [10] for SWCNT binding, ensuring maximum loading assumed for PX-866/SWCNT cell viability assays. SWCNTs dispersion with active agents was finally followed by the addition of 1600 µM of DSPE-PEG 5000 (NanoCS, Boston, MA, USA) attached to SWCNTs via additional ultrasonic agitation with Covaris at 50 W for 6 min. SWCNT concentration in the samples was matched via absorption measurements with an extinction coefficient of 31.25 mL mg^−1^cm^−1^ at 632 nm [10].

Transmission electron microscopy (TEM JEOL JEM-2100) was utilized to observe the morphology of final SWNT formulations. The sample for TEM measurement was prepared on a carbon-coated 200-mesh copper grid under ambient conditions. 

### 3.2. Optical Characterization

Fluorescence and absorbance spectra were measured using an NS2 NanoSpectralyzer (Applied NanoFluorescence, Houston, TX, USA). To measure the photoluminescence of SWCNTs, a 637-nm diode laser excitation was used. The collected fluorescence spectra were simulated using fully integrated Nanospectralyzer GlobalFit software. The software allows for the simulation of experimental SWCNT spectra with individual chirality SWCNT emission peaks for spectra with four excitations, models excitation–emission maps based on those, and extracts relative emissive SWCNT chirality abundances in the sample.

SWCNTs concentration was calculated using the absorbance spectrum similarly to [77,78]:

SWCNTs Concentration (mg/mL) = Absorbance at 632 nm/31.25 mL mg^−1^cm^−1^ (Experimental extinction co-efficient).

### 3.3. Fluorescence Microscopy Measurements

Fluorescence microscopy was performed using an Olympus IX73 fluorescence microscope with 60× (IR-corrected Olympus Plan Apo, Japan) water immersion objective coupled to two detectors: spectrally filtered by 10 filters throughout the visible Hamamatsu Image EMCCD camera, and InGaAs Xenics (Xeva-7870, XEN-000110) coupled to a hyperspectral fluorescence imager (Photon etc., Montreal, QC, Canada) This allowed for spectrally-resolved imaging both in the visible and near-infrared.

### 3.4. Imaging in the Visible Region

We imaged the green (535 nm) emission of PX-866 in vitro with lamp excitation and (375 ± 25 nm) excitation and (535 ± 20 nm) emission filters by first determining the integration and lamp intensity settings that resulted in zero autofluorescence emission from non-treatment control cells and using the corresponding settings for PX-866 fluorescence imaging.

### 3.5. Imaging in the NIR Region

SWCNTs fluorescence in hepatocellular carcinoma (HepG2) and HeLa cells was imaged with an InGaAs camera (Xenics Xeva, Belgium) and an NIR hyperspectral imager (Photon etc. IMA-IR^TM^) with 637 nm (130 mW output power) diode laser excitation. This infrared hyperspectral imager captures full spatial information simultaneously utilizing a Bragg grating imaging filter [79] which collects spectral information successively providing spectrally resolved imaging. Individual SWCNT fluorescence could be resolved for all emissive chiralities using band-pass filtering mode (950–1450 nm), but only particular SWCNT chiralities can be imaged by selecting a specific spectral region. For (7,5) and (7,6) SWCNT chiralities dispersed with PX-866 and CCR5 siRNA, 1030 and 1130 nm filter positions were found optimal. In-vitro control images without SWCNTs were also captured, ensuring no emission in the NIR region. 

### 3.6. Cell Culture

In this work, we used liver hepatocellular carcinoma (HepG2) and HeLa cell lines maintained in a Thermo-Scientific Midi 40 CO_2_ Incubator at 37.1 °C with 5% carbon dioxide and 95% air. In order to prepare the glass coverslips for microscopy imaging, they were placed at the bottom of 6-well plates followed by adding cells in the media. SWCNT-carried formulations were added at a concentration of 2 µg/mL in each well after 4 hours of cell attachment to the coverslips. Cells were further washed with 0.5 mL of PBS (phosphate-buffered saline) to remove extracellular SWCNTs, followed by fixing them with 4% paraformaldehyde at room temperature for 30 min. After that, the cell samples were rewashed with 0.5 mL of PBS for the microscopy imaging. Transfection points of 0.5, 1, 3 h were used for imaging, with 2 µg/mL SWCNTs added to each well. 

### 3.7. MTT Assays

In order to assess the cytotoxicity for SWCNT, PX866, and their complex, HepG2 (human hepatocellular carcinoma) and HeLa cells were plated in a 96-well plate at a density of 5000 cells per well (100 μL/well) and kept in an incubator overnight at 37.1 °C while maintaining the CO_2_/air ratio of 1:19. After 24 h of incubation, the samples (PX-866, SWCNTs, or SWCNT/PX-866 formulations) were added into each well at concentrations ranging from 0.125 to 2.5 µg/mL. After 24 h of incubation, the medium was replaced by 100 μL of 1 mg/mL thiazolyl blue tetrazolium bromide. The cells were incubated further for 4 h followed by the replacement of MTT (3-(4–dimethylthiazol-2-yl)-2,5-diphenyltetrazolium bromide) with 100 μL of DMSO (dimethyl sulfoxide) in order to solubilize the precipitation. Reduction in MTT influences the metabolic activity of living cells, which can be assessed with absorbance measurements since living cells metabolize the MTT and form a highly absorbing purple colored byproduct known as formazan [80]. We measured the absorbance (essentially the cell viability) of the final sample at 540 nm wavelength using the FLUOstar Omega microplate reader.

### 3.8. siRNA Transfection

The day before transfection, HepG2 cells were seeded onto 24-well plates in DMEM medium with 10% FBS to give ~30% confluence at the time of transfection. Before the transfection, the culture medium was replaced with fresh DMEM medium supplemented with 15% FBS and the SWCNTs complexed with CCR5 siRNA and DSPE-PEG 5000 as described above were then added to the medium, after which the cells were cultured for 48 h. For transfection efficiency examination, flow cytometry assay was performed at 48 h post-treatment.

### 3.9. Flow Cytometry

For the flow cytometry of the transfection efficiency experiment, HepG2 cells were washed twice with PBS and harvested by trypsin/EDTA. Following trypsinization, the cells were washed by centrifugation and resuspended in staining buffer (1 × PBS, 2% FBS, 0.5% EDTA, and 0.1% NaN_3_). The cell suspension was stained with PE (phycoerythrin)-conjugated human anti-CCR5 antibody (Biolegend, Cat#359105) for 30 min on ice. To test the unspecific antibody binding, IgG (BioLegend, Cat#359105) was used as the isotype control. Flow cytometry was performed using an Accuri C6 plus flow cytometer (BD Biosciences, San Jose, CA, USA), and the data were analyzed using FlowJo software (www.flowjo.com/solutions/flowjo).

### 3.10. Image Analysis

We utilized ImageJ software to analyze all the images, including the subtraction of the backgrounds and overlays of the bright-field cell images with emission from PX-866 and two sorted SWCNT chiralities (i.e., (7,5), (7,6)) at 1030 and 1130 nm, respectively.

## 4. Conclusions

In this work, we developed and tested a single-walled carbon nanotube-based drug/gene combination therapeutic platform that allows for the image-tracking of each therapeutic agent. Chirality-sorted SWCNTs emitting at different wavelengths in the NIR were used to selectively track and deliver two therapeutic payloads in vitro: CCR5 siRNA and the small-molecule drug PX-866, targeting inflammation and fibrosis factors that mediate the translation of nonalcoholic steatohepatitis into hepatocellular carcinoma. For that purpose, (7,5) and (7,6) chiral SWCNTs separated from raw CoMoCAT starting material via aqueous two-phase extraction with over ~40% fluorescence-derived purities and cleared from toxic sorting surfactants were individualized by the dispersion and non-covalent complexation with the corresponding drug or gene. Hyperspectral NIR and spectrally-resolved visible fluorescence imaging allowed simultaneous monitoring at 1030 nm emission of (7,5) SWCNTs carrying PX-866 and at 1130 nm emission of (7,6) SWCNTs complexed with CCR5 siRNA internalized in HepG2 cells, as well as the visible 528 nm emission from PX-866 as it was released from the SWCNTs inside the cells. This work demonstrates: (1) the successful delivery of drug/gene therapy with sorted chiral SWCNTs; (2) the potential for locating each therapeutic agent separately through characteristic SWCNT fluorescence; and (3) the improved efficacy of both therapeutics when delivered with SWCNTs with substantially increased effect of SWCNT/PX866 over PX866 alone and high (over 90%) apparent knockdown of CCR5 siRNA when carried by SWCNTs, suggesting a promising potential of these formulations for combination NASH therapy. The emission of individual chiral SWCNTs in the near-infrared with low autofluorescence and high penetration depth could be utilized for multi-gene/drug delivery and imaging in animal models, whereas the high non-targeted liver accumulation of SWCNTs makes them advantageous candidates for liver conditions such as NASH. 

## Supplementary Materials

The following are available online at https://www.mdpi.com/2072-6694/11/8/1175/s1. Figure S1: Fluorescence and corresponding absorbance spectra of Parent SWCNT sample: raw SWCNTs dispersed with DOC/PEG/Dextran that are used as a starting material for ATPE sorting, bottom phases of ATP system with 3 mg/mL and 4 mg/mL SDS added to sort top phases containing (7,5) and (7,6) chirality-sorted SWCNTs at 3 mg/mL and 4 mg/mL SDS, respectively, Figure S2: Fluorescence and corresponding absorbance spectra of SWCNT collected from top phases containing (7,5) and (7,6) chirality-sorted SWCNTs at 3 mg/mL and 4 mg/mL SDS, respectively, Figure S3: Table describing the peak positions and peak shifts for the following samples: Sample-1: SWCNTs + sorting surfactants; Sample-2: SWCNTs + siRNA; Sample-3: Washed/annealed SWCNTs + siRNA. The oval shaped marker denotes the full restoration of the peak positions after the centrifugal washing/annealing and siRNA dispersion to those of raw SWCNTs dispersed with sRNA, whereas rectangle marker depicts partial but significant peak position recovery after washing/thermal annealing, Figure S4: Fluorescence spectra of untreated (no thermal annealing) SWCNTs/EGFR siRNA formulations, and thermally annealed SWCNTs dispersed with EGFR siRNA, Figure S5: Comparison between the fluorescence spectra of washed/annealed SWCNTs + siRNA before and after the centrifugation, Figure S6: Absorbance spectra of (7,5) SWCNT/PX-866, and (7,6) SWCNT/CCR5 siRNA, Figure S7: Fluorescence spectra of only PX-866 and PX-866+SWCNT showing the quenching of PX-866 fluorescence after the loading of PX-866 on the SWCNTs, Figure S8: TEM images of the mixture of (7,5) SWCNTs/DSPE-PEG/siCCR5 and (7,6) SWCNTs/DSPE-PEG/PX-866 showing SWCNTs coating (DSPE-PEG-5000, and gene or drug), Figure S9: Fluorescence images of non-treatment control (without SWCNTs), Parent CoMoCat SWCNTs in aqueous dispersion with ATPE surfactants containing SWCNTs of various chiralities imaged at 1030 nm corresponding to (7,5) SWCNT emission, 1130 nm corresponding to (7,6) SWCNT emission, and Fluorescence overlay image of both (7,5) and (7,6) SWCNTs in the sample, Figure S10: Brightfield/fluorescence overlay images of cellular (HepG2 cells) uptake of (7,5) SWCNTs/PX-866 imaged at 1030 nm, (7,6) SWCNTs/siRNA imaged at 1130 nm, and px-866 released from SWCNTs imaged at 535 nm, Figure S11: Brightfield/fluorescence overlay images of cellular (HepG2 cells) uptake and release of px-866 from SWCNTs with 1, 3, 4.5, 12 h incubation time imaged at 535 nm, Figure S12: Brightfield/ NIR fluorescence overlay images of cellular (HeLa cells) uptake of SWCNTs/siRNA imaged with 637 nm laser excitation, Figure S13: MTT assay cell viability of HeLa cells treated with SWCNT/siRNA.
