# Supplementary material for: Multi-Drug/Gene NASH Therapy Delivery and Selective Hyperspectral NIR Imaging Using Chirality-Sorted Single-Walled Carbon Nanotubes
Source: Cancers (Basel). 2019 Aug 14;11(8):1175. doi: 10.3390/cancers11081175 (PMC6721580; doi:10.3390/cancers11081175)
Supplement: Supplementary file 1 [file cancers-11-01175-s001.pdf]

Supplementary Materials: Multi-Drug/Gene NASH Therapy Delivery and Selective Hyperspectral Near-IR Imaging Using Chirality-Sorted Single-Walled Carbon Nanotubes

Md Tanvir Hasan, Elizabeth Campbell, Olga Sizova, Veronica Lyle, Giridhar Akkaraju, D. Lynn Kirkpatrick and Anton V. Naumov

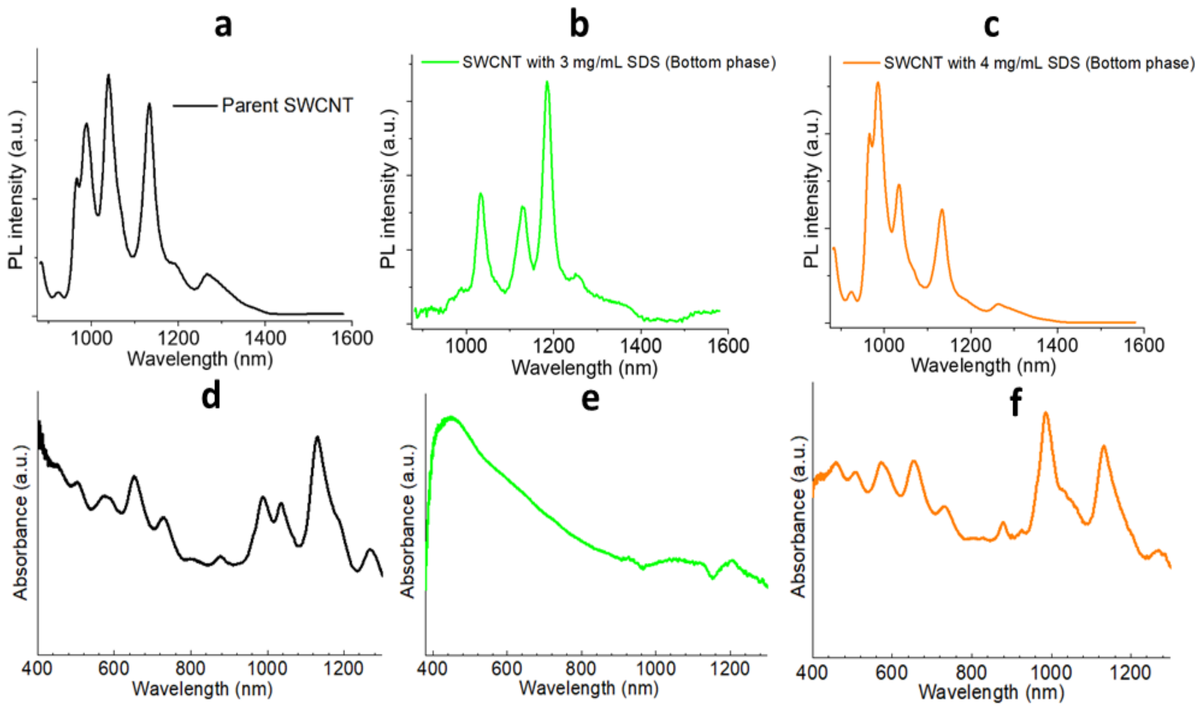

Figure S1. Fluorescence spectra of (**a**) Parent SWCNT sample: raw SWCNTs dispersed with DOC/PEG/Dextran that are used as a starting material for ATPE sorting, bottom phases of ATP system with (**b**) 3 mg/mL and (**c**) 4 mg/mL SDS added to sort top phases containing (7,5) and (7,6) chirality-sorted SWCNTs at 3 mg/mL and 4 mg/mL SDS, respectively. Corresponding absorbance spectra of (**d**) Parent SWCNT sample, bottom phases of ATP system with (**e**) 3 mg/mL and (**f**) 4 mg/mL SDS added to sort top phases.

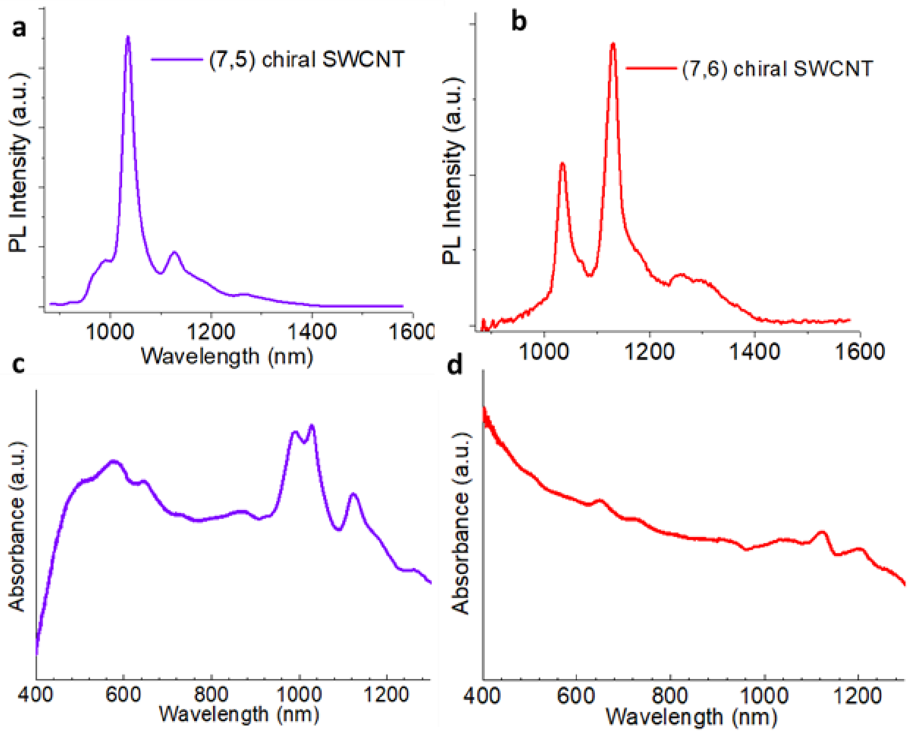

Figure S2. Fluorescence spectra of SWCNT collected from top phases containing (**a**) (7,5) and (**b**) (7,6) chirality-sorted SWCNTs at 3 mg/mL and 4 mg/mL SDS, respectively. Corresponding absorbance spectra of (**c**) (7,5) and (**d**) (7,6) chirality-sorted SWCNTs at 3 mg/mL and 4 mg/mL SDS, respectively.

Corresponding chirality: (n,m) Species	SWCNTs emission (Em) features	SWCNTs+ sorting surfactants (sample - 1)	SWCNTs+ siRNA (sample - 2)	Red Shifts in peak positions in sample-2 w.r.t sample-1 (nm)	Washed/annealed SWCNTs +siRNA (sample - 3)	Red Shifts in peak positions in sample-3 w.r.t sample-1 (nm)
(6,5)	Peak position (nm)	966	974	8	974	8
(7,3)		986	996	10	1004	18
(7,5)		1035	1041	6	1046	11
(7,6)		1130	1141	11	1141	11
(11,3)		1185	1213	28	1212	27
(11,1)		1265	1315	50	1308	43

Figure S3. Table describing the peak positions and peak shifts for the following samples: Sample-1: SWCNTs + sorting surfactants; Sample-2: SWCNTs + siRNA; Sample-3: Washed/annealed SWCNTs + siRNA. The oval shaped marker denotes the full restoration of the peak positions after the centrifugal washing/annealing and siRNA dispersion to those of raw SWCNTs dispersed with siRNA, whereas rectangle marker depicts partial but significant peak position recovery after washing/thermal annealing.

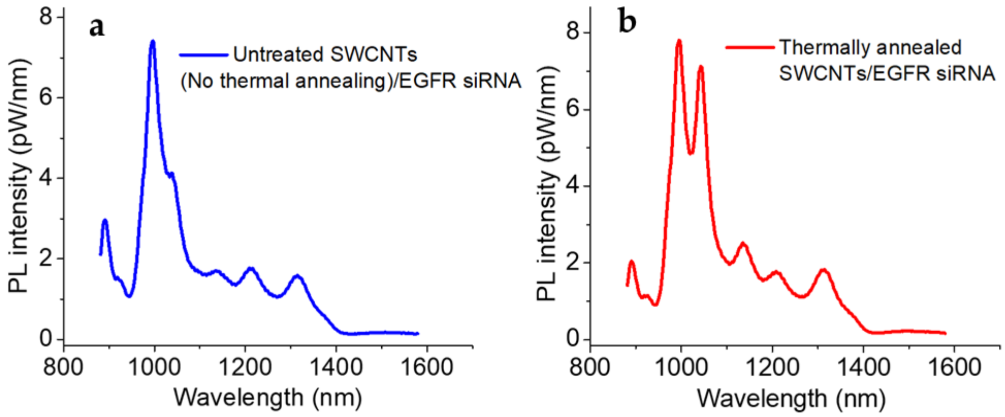

Figure S4. Fluorescence spectra of (**a**) untreated (no thermal annealing) SWCNTs/EGFR siRNA formulations, and (**b**) thermally annealed SWCNTs dispersed with EGFR siRNA.

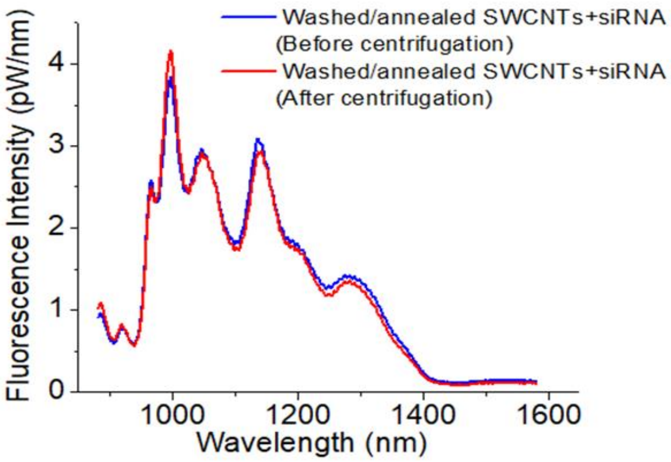

Figure S5. Comparison between the fluorescence spectra of washed/annealed SWCNTs+siRNA before and after the centrifugation.

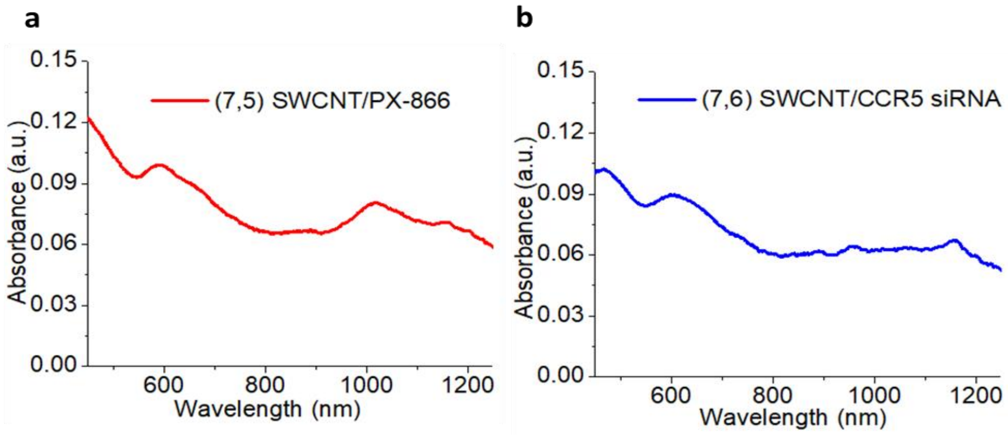

Figure S6. Absorbance spectra of (**a**) (7,5) SWCNT/PX-866, (**b**) (7,6) SWCNT/CCR5 siRNA showing weak peaks in the absorbance spectrum which could be potentially caused by blending in with absorption backgrounds [1].

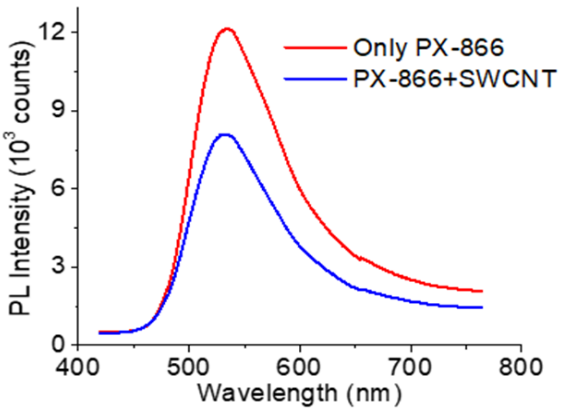

Figure S7. Fluorescence spectra of only PX-866 and PX-866+SWCNT showing the quenching of PX-866 fluorescence after the loading of PX-866 on the SWCNTs.

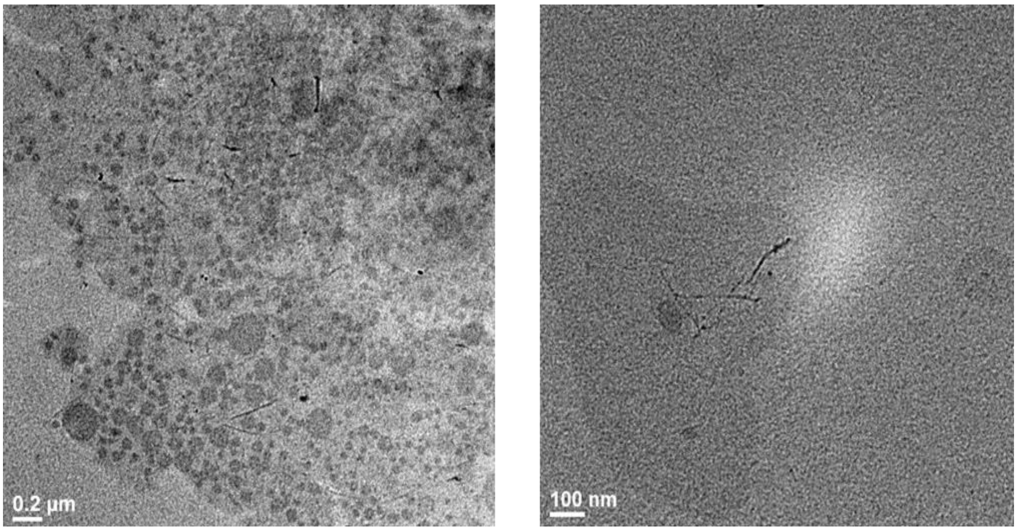

Figure S8. TEM images of the mixture of (7,5) SWCNTs/DSPE-PEG/siCCR5 and (7,6) SWCNTs/DSPE-PEG/PX-866 showing SWCNTs coating (DSPE-PEG-5000, and gene or drug).

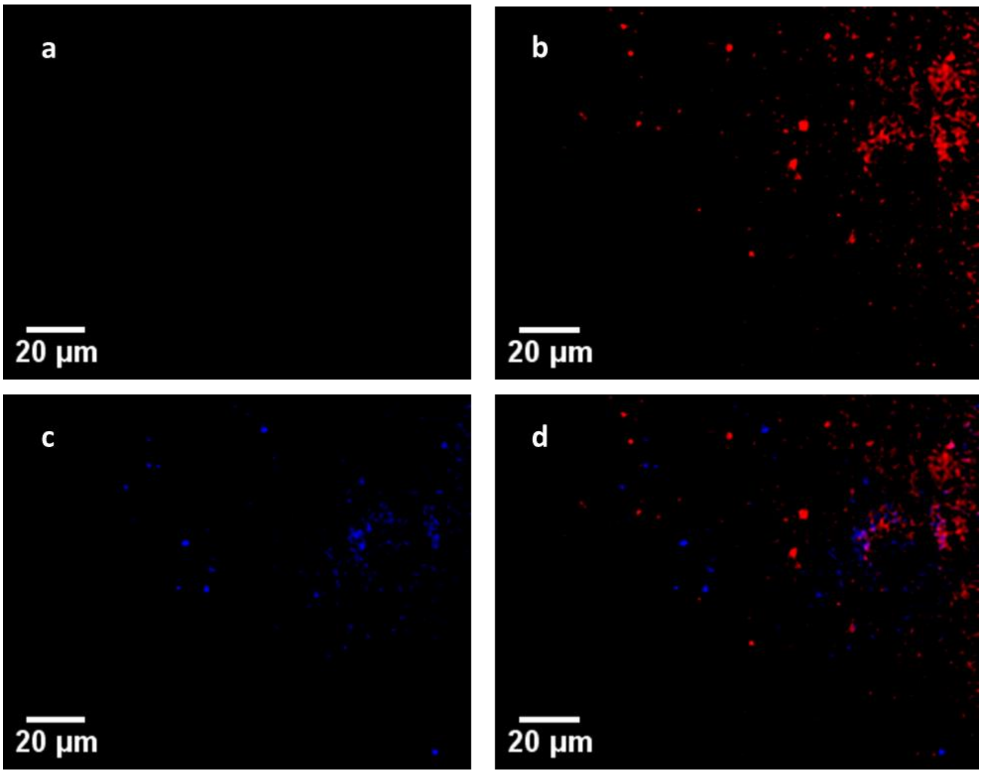

Figure S9. Fluorescence images of (**a**) non-treatment control (without SWCNTs). Parent CoMoCat SWCNTs in aqueous dispersion with ATPE surfactants containing SWCNTs of various chiralities imaged at (**b**) 1030 nm corresponding to (7,5) SWCNT emission (**c**) 1130 nm corresponding to (7,6) SWCNT emission. (**d**) Fluorescence overlay image of both (7,5) and (7,6) SWCNTs in the sample.

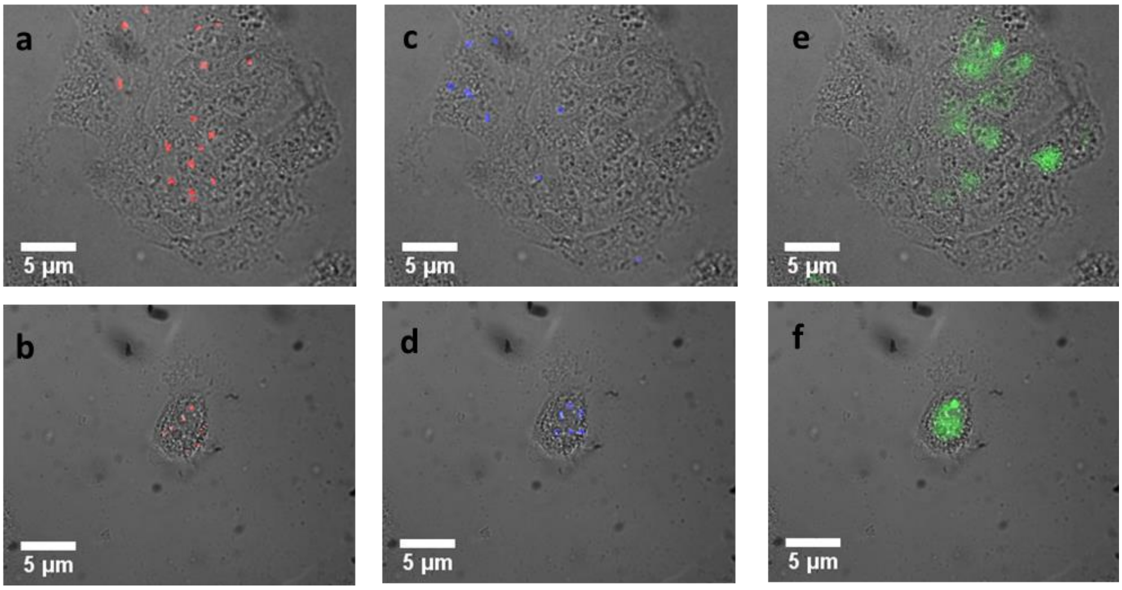

Figure S10. Brightfield/fluorescence overlay images of cellular (HepG2 cells) uptake of (**a**,**b**) (7,5) SWCNTs/PX-866 imaged at 1030 nm (**c**,**d**) (7,6) SWCNTs/siRNA imaged at 1130 nm. (**e**,**f**) px-866 released from SWCNTs imaged at 535 nm.

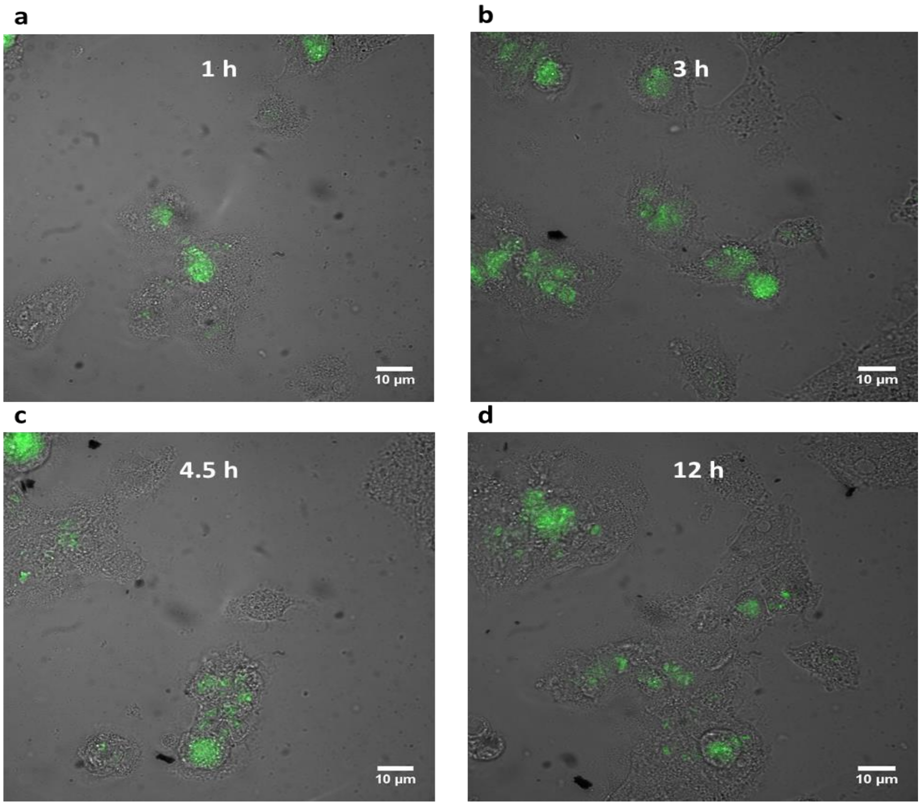

Figure S11. Brightfield/fluorescence overlay images of cellular (HepG2 cells) uptake and release of px-866 from SWCNTs with (**a**) 1h, (**b**) 3h, (**c**) 4.5h, and (**d**) 12h incubation time imaged at 535 nm.

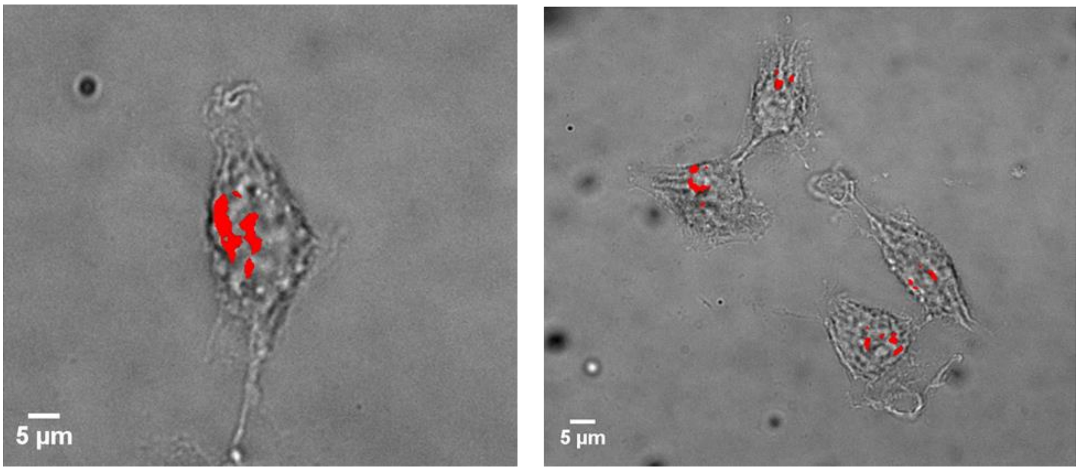

Figure S12. Brightfield/ NIR fluorescence overlay images of cellular (HeLa cells) uptake of SWCNTs/siRNA imaged with 637 nm laser excitation.

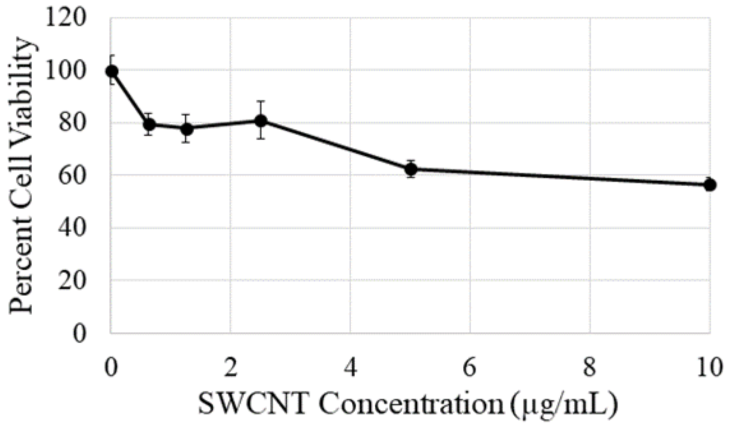

Figure S13. MTT assay cell viability of HeLa cells treated with SWCNT/siRNA conjugate.

References

1. Naumov, A.V.; Ghosh, S.; Tsyboulski, D.A.;Bachilo, S.M.; Weisman R.B. Analyzing Absorption Backgrounds in Single-Walled Carbon Nanotube Spectra. *A. C. S. Nano.*, **2011**, **5**(3), 1639–1648.

© 2019 by the authors. Licensee MDPI, Basel, Switzerland. This article is an open access article distributed under the terms and conditions of the Creative Commons Attribution (CC BY) license (http://creativecommons.org/licenses/by/4.0/).
